# Comparison of W14 fastening system variants depending on clamping force

**DOI:** 10.1038/s41598-025-09483-0

**Published:** 2025-07-10

**Authors:** Łukasz Chudyba, Tomasz Lisowicz, Piotr Piech, Przemysław Stanisławski

**Affiliations:** 1 Faculty of Civil Engineering, Cracow University of Technology, Krakow, Poland; 2 Track Tec S.A., Katowice, Poland

**Keywords:** Fastening system W14, Laboratory research of fastening system, Clamping force, FEM, Engineering, Materials science

## Abstract

This paper presents a comparative analysis of the impact of individual elements of the W14 fastening system on clamping force. Clamping force in the context of railway fastening systems refers to the force that holds the rail firmly against the sleeper to ensure track stability and maintain proper rail alignment under dynamic loads. The results of laboratory tests carried out in accordance with the applicable European standards of the PN-EN 13,481 and PN-EN 13,146 series are described synthetically. Actual clamping force achieved is highly dependent on the height of the rail pad, as the applied torque can result in different clamping forces depending on this factor. It is important to note that the critical parameter for the fastening system’s performance is not the applied torque itself, but rather the resulting clamping force. This force is directly influenced by the rail pad height, as well. as other factors such as the static stiffness of the pad. Therefore, achieving the appropriate clamping force requires careful consideration of these factors, as they significantly impact the system’s stability and overall efficiency.

## Introduction

The elastic fastening system is a set of mutually cooperating elements that enable the rail to be attached to the support in the required position, allowing for intentional displacement (vertical, transverse, and longitudinal) while maintaining the rail’s attachment. In elastic fastening systems, the rail is fastened using a properly shaped clamp, which ensures that stresses are maintained at a constant level, even when loaded by rolling stock wheels. The primary functions of the fastening system are.

^1–3^:

(1) Transferring forces from the rails to the supporting element (e.g., sleeper),

(2) Damping vibrations and impacts caused by the movement of rail vehicles,

(3) Maintaining the appropriate track gauge,

(4) Maintaining the appropriate lateral inclination of the rails,

(5) Ensuring proper clamping of the rail to the supporting element to limit longitudinal displacements,

(6) Providing electrical insulation between rail tracks,

(7) Ensuring adequate track stiffness in the horizontal plane (resistance to rail rotation relative.

(8) to the sleeper),

(9) Ensuring adequate track stiffness in the vertical plane (resistance to rail twisting relative.

(10) to the longitudinal axis).

The selection of the fastening system and its constituent elements within the entire assembly plays a crucial role in the correct and reliable functioning of the track. It is necessary to use rail pads with the required clamping force and rail pads with such stiffness that minimizes their impact on ballast degradation, as well as reducing vibrations resulting from railway track operation. Due to the provision of rail deflection on an elastic pad, stresses are distributed over a greater number of sleepers. The overall stiffness of the track is influenced by the resistance to rail rotation and the inverse value of the fastening stiffness coefficient.The value of the moment M, resulting from the action of the vertical force Q and the transverse force Y, is calculated by Eq. (1) according to^4^:

1 $$\:M={c}_{T}\cdot\:\:\varphi\:=(\frac{{b}^{2}\cdot\:{c}_{stat}}{12}+\frac{{c}^{2}\cdot\:{c}_{clip}}{2})\cdot\:\varphi\:$$

In which $\:{c}_{T}$ is the rail rotation resistance value $\:\left[\frac{kNm}{^\circ\:}\right]$, $\:\varphi\:$ is the rail rotation angle [˚], $\:{c}_{stat}$ is the static stiffness of the rail pad $\:\left[\frac{kN}{cm}\right]$, $\:{c}_{clip}$ is the stiffness of the rail clamp $\:\left[\frac{kN}{cm}\right]$, $\:b$ is the width of the rail pad [cm], *c* is the distance between rail clamps [cm].

The transverse resistance value of the fastening system, resulting from the action of the transverse force, is calculated using Eq. (2) according to^4^:

2 $$\:H={c}_{H}\cdot\:{s}_{2}$$

Where $\:{c}_{H}$ is the inverse stiffness value $\:\left[\frac{kN}{mm}\right]$, $\:{s}_{2}$ is the horizontal displacement of the rail foot relative to the sleeper $\:\left[mm\right]$. The vertical reaction value is calculated using Eq. (3) according to^4^:

3 $$\:{F}_{z}={c}_{z}\cdot\:z=\left({c}_{stat}-2\cdot\:{c\:}_{clip}\right)\cdot\:z$$

Where $\:{c}_{z}$ is the vertical stiffness of the fastening system $\:\left[\frac{kN}{cm}\right]$, z is the vertical elasticity [cm].

**Fig. 1:**
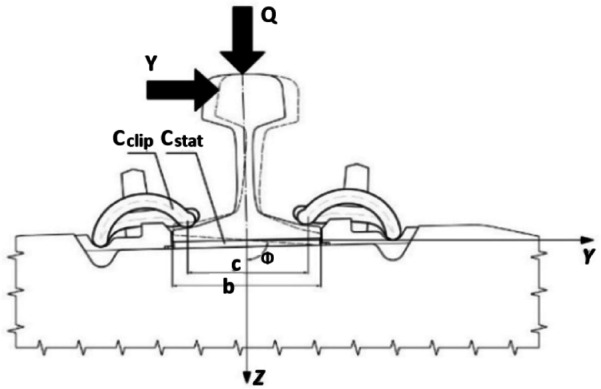
illustrates the schematic representation of the forces acting on the anchoring node.

Figure 1. The diagram illustrates the forces acting transversely and vertically on the fastening system^4^.

In this paper, we aim to understand the mechanism of change in clamping force values in the case of various variants of the W14 fastening system, as well as to identify potential causes of this phenomenon. We hope that gaining insight into this mechanism will lead to improved design practices for railway fastening system components. In papers^5–7^, the influence of constituent elements on various types of fastening systems has been elucidated. Due to the intricate and nonlinear nature of the fastening system’s operation, as depicted in^8–13^, precise observations solely through.

real-world testing are not feasible. Consequently, a nonlinear Finite Element Analysis (FEA) approach was employed. The fastening system selected for this study includes rail pads that have been used for many years in European railways. The pads were chosen to differ in both thickness and static stiffness, as these parameters may influence the clamping force.

This paper is part of a broader research series devoted to successive variants of W-type rail fastenings. At the present stage we concentrate on how the vertical clearance **x**—measured between the upper edge of the angular guide plate (Wfp) and the top surface of the rail pad—governs the clamping force, with explicit consideration of the pad’s static stiffness and the screw-tightening torque. In later instalments we will examine the joint influence of **x** and static stiffness on other critical fastening-system parameters, such as longitudinal resistance and impact-load damping. The ultimate aim of the programme is to pinpoint an optimised fastening solution that can be deployed reliably on a wide variety of railway lines.

## Components of the W14 fastening systems utilized in the analyses

For comparing the clamping force values depending on the type of rail pad and rail clamp, the following components of the W14 fastening system were selected for the study: SKL 14 rail clamps, SKL 14RT rail clamps, angular guiding plates Wfp 14K12; rail pads Zw 687a, Zw 700, Zw 900, Zw 1000; dowels Sdu 25; screws Ss 35^2^.

**Fig. 2:**
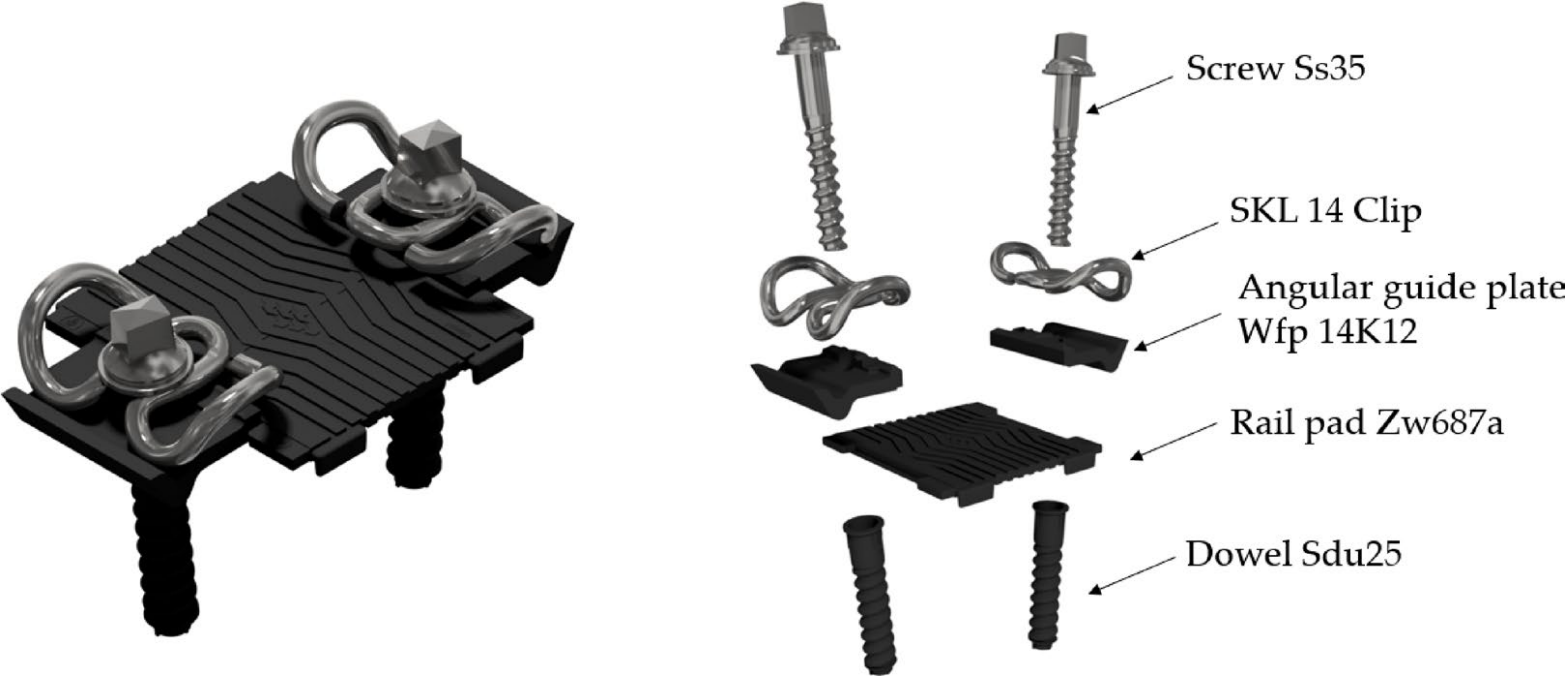
The W14 fastening system.

Figure 2 illustrates the standard solution of the W14 fastening system.

**Fig. 3:**
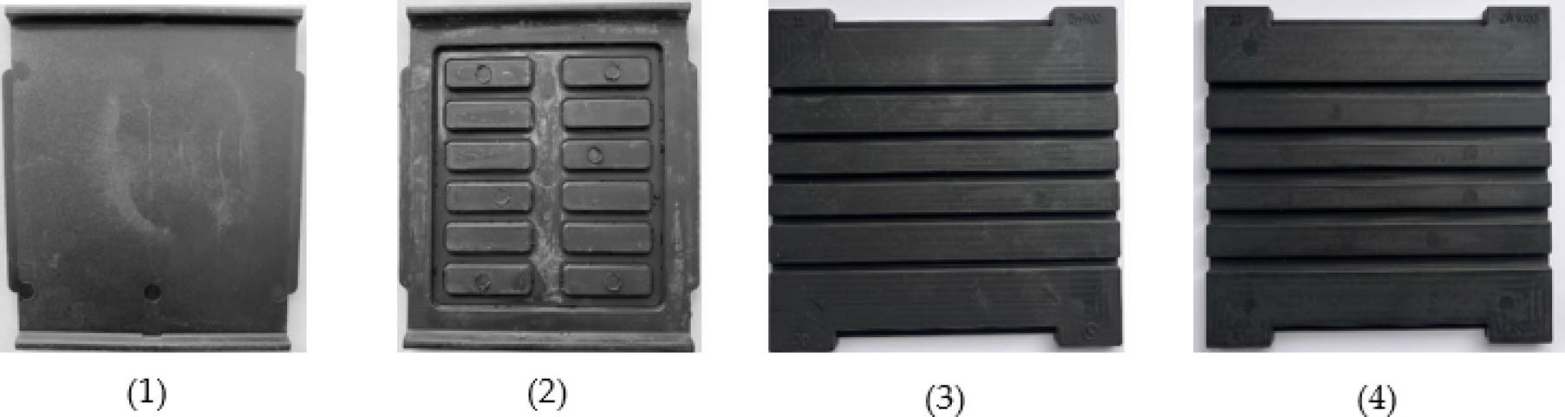
Rail pads used for comparative studies: (1) Zw 687a, (**2**) Zw 700, (**3**) Zw 900, (**4**) Zw 1000.

In Fig. 3, the analysed rail pads are depicted, while Table 1 presents the parameters of the analysed rail pads.

**Table 1:** Parameters of the analysed rail pads.

No.	Rail Pad Type	Thickness	Static Stiffness k_SP_	Material
1	Zw 1000	10 [mm]	40 [kN/mm]	Natural Rubber NR/BR
2	Zw 700	7 [mm]	70 [kN/mm]	Natural Rubber NR/BR
3	Zw 687a	6 [mm]	640 [kN/mm]	EVA
4	Zw 900	9 [mm]	75 [kN/mm]	Natural Rubber NR/BR

Two types of rail clamps were also utilized in the study: SKL 14 and SKL 14 RT. It can be assumed that the clamps have the same shape (the same guiding curve), differing only in the thickness of the.

cross-section of the rod from which they are made, respectively: from a rod with a diameter of Ø 13.2 mm and Ø 13.5 mm.

## Investigated variants of the W14 fastening system

**Fig. 4:**
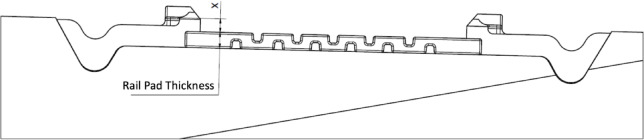
Height difference between the rail pad and the contact point of the SKL 14 rail clamp on the angular guiding plate Wfp - dimension x.

From the presented components used in the W14 fastening systems, several variants of complete assemblies were prepared to compare the clamping force. Rail pads were selected to differ in thickness and static stiffness k_SP_, which may affect the change in the thickness of the rail pad when pressed by the rail after the rail clamps were installed with the appropriate screw tightening torque. The variants of W14 fastening system configurations used for analysis along with dimension x is listed in Table 2. Figure 4 defines the difference in rail pad height, with the location of the rail clamp’s.

support - dimension x.

**Table 2:** Analysed variants of W14 fastening systems with descriptions.

Variant name	Rail Pad	Static stiffness k_SP_ [kN/mm]	Angular guiding plate Wfp	Rail Clamp	Dimension x
I a	Zw 1000	40	Wfp 14K12	Skl 14	7
I b	Zw 1000	40	Wfp 14K12	Skl 14RT	7
II a	Zw 700	85	Wfp 14K12	Skl 14	10
II b	Zw 700	85	Wfp 14K12	Skl 14RT	10
III a	Zw 687a	642	Wfp 14K12	Skl 14	11
III b	Zw 687a	642	Wfp 14K12	Skl 14RT	11
IV a	Zw 900	90	Wfp 14K12	Skl 14	8
IV b	Zw 900	90	Wfp 14K12	Skl 14RT	8

All laboratory tests were conducted on a PS-93 W14 prestressed concrete sleeper with a 60E1 rail having a foot width of 150 mm. The tests were performed at the accredited Building Materials and Structures Research Laboratory of the Cracow University of Technology in accordance with standards^14–16^.

**Fig. 5:**
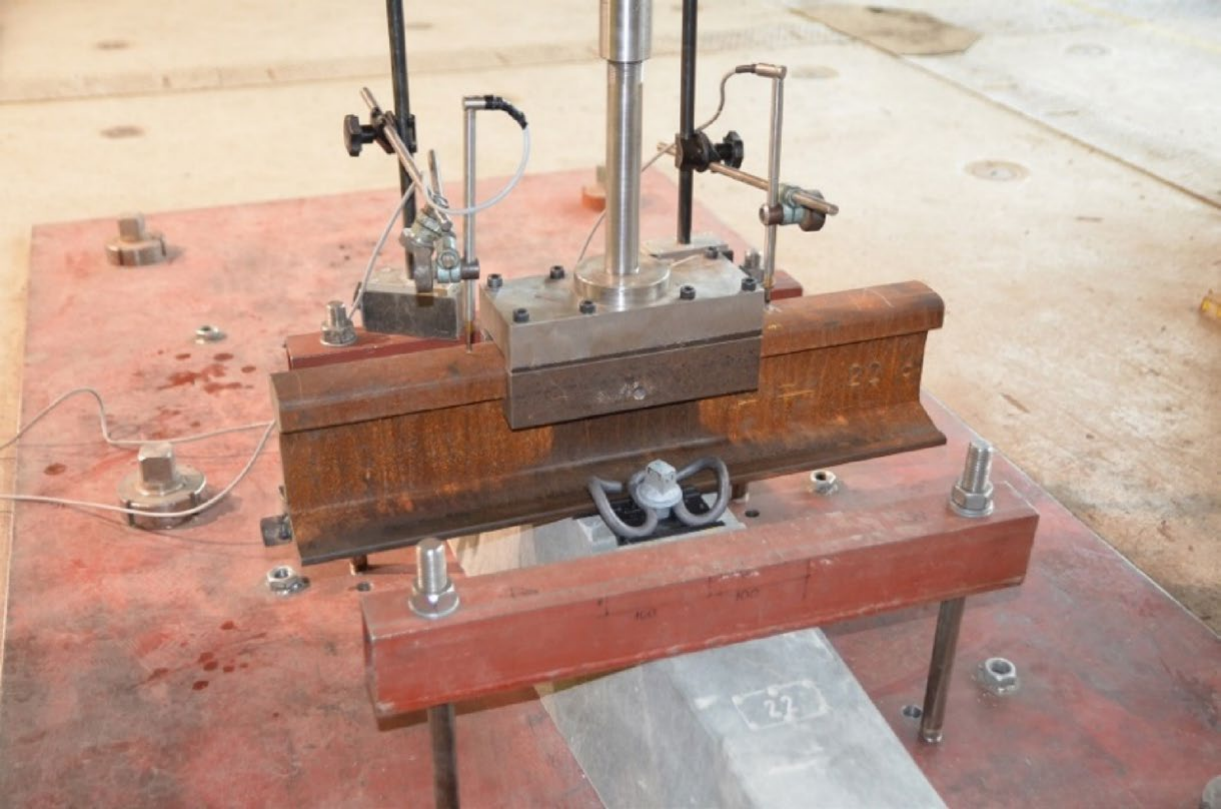
Clamping force testing setup.

The aim of the research was to examine the influence of the height difference of the rail pad, the angular plate, and the screw tightening torque on the clamping force of the W14 rail fastening system. Figure 5 depicts the clamping force testing setup.

The experiment was conducted according to norm PN-EN 13146-7. The general steps are as follows:

1. Assembly of the system including appropriate torque on the screws.
2. Application of the vertical force to the rail until there is no more clamp between rail and rail pad.
3. Pulling out the rail pad of the assembly.
4. Gradually release force to the moment when rail seat on the sleeper.
5. Read force at the moment where the rail was at the initial position after assembly.

## Experimental results

For each variant, the fastening assembly was installed on a PS-93 W prestressed concrete sleeper fitted with a 1 m-long rail section. Four inductive displacement sensors were fixed to the rail web (Fig. 5) to record the vertical movement of the rail during the lift-off test. The screws were tightened to three torque levels—200, 220 and 250 Nm—so that the influence of tightening torque on clamping force could be evaluated.

**Fig. 6:**
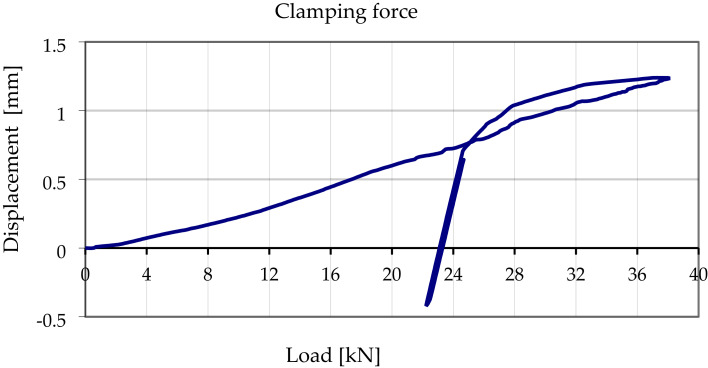
Example graph of displacement versus load during clamping force testing for rail pad Zw 1000 and rail clamp SKL 14 RT - variant IB.

After tightening, an increasing tensile load P was applied to the rail while keeping the rail base strictly parallel to the rail seat (no tilting). The load was raised until the rail pad could be removed. The pad was then taken out and the load gradually reduced until the mean reading of the four displacement sensors returned to zero; this load was noted as P. Next, the load was lowered to approximately 0.9 P. Maintaining a loading rate not exceeding 10 kN min⁻¹, the tensile force was once more increased—this time to 1.1 P—while the average vertical displacement continued to be recorded. From the resulting load–displacement curve (Fig. 6) the value of P₀ at zero displacement was extracted and taken as the clamping force.

**Fig. 7:**
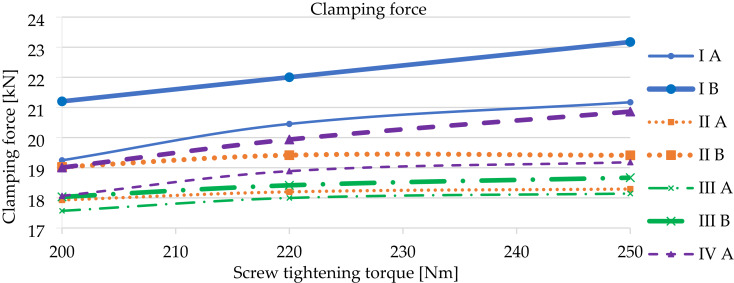
Average clamping force values depending on the screw tightening torque. Source: Authors’ own work.

In total, three such measurements were carried out for every investigated variant and torque level, in accordance with standard^15^. Figure 6 presents a representative load–displacement trace for variant Ib (rail pad Zw 1000 with clamp SKL 14 RT), while Fig. 7 compares the resulting mean clamping forces as a function of tightening torque and the height offset x.

From the obtained results of the laboratory tests, it can be concluded that the highest clamping force values for each variant pair, depending on the type of rail clamp, were obtained for SKL 14 RT at a tightening torque of 250 Nm. It was observed that the clamping force increases with a decrease in the dimension x, indicating an increase in the rail pad height. Additionally, a decrease in the static stiffness of the rail pad (k_SP_) contributes to an increase in clamping force. This is a result of the greater initial displacement of the rail pad during screw tightening.

**Fig. 8:**
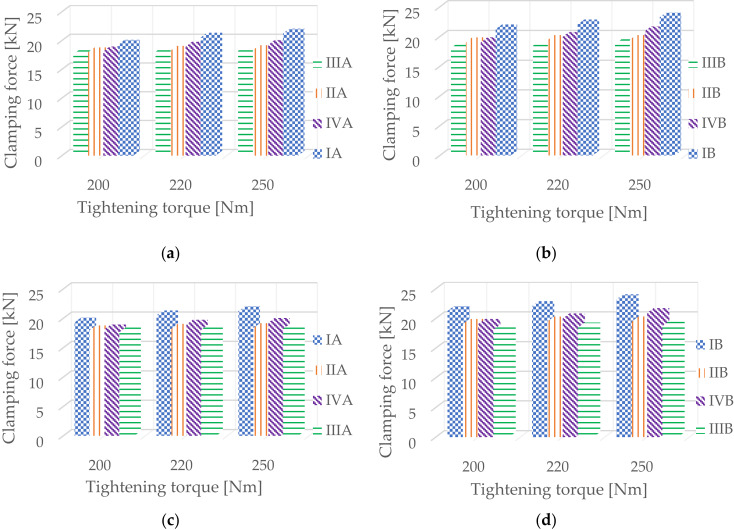
compares the results depending on the thickness or stiffness of the rail pad.

Figure 8. Clamping force values a), b) for changes in rail pad thickness and c), d) for changes in static stiffness.

The research results presented in Fig. 8a and b indicate an increase in clamping force with increasing rail pad thickness, and consequently, a decrease in dimension x.

## Calculations using the finite element method (FEM)

**Fig. 9:**
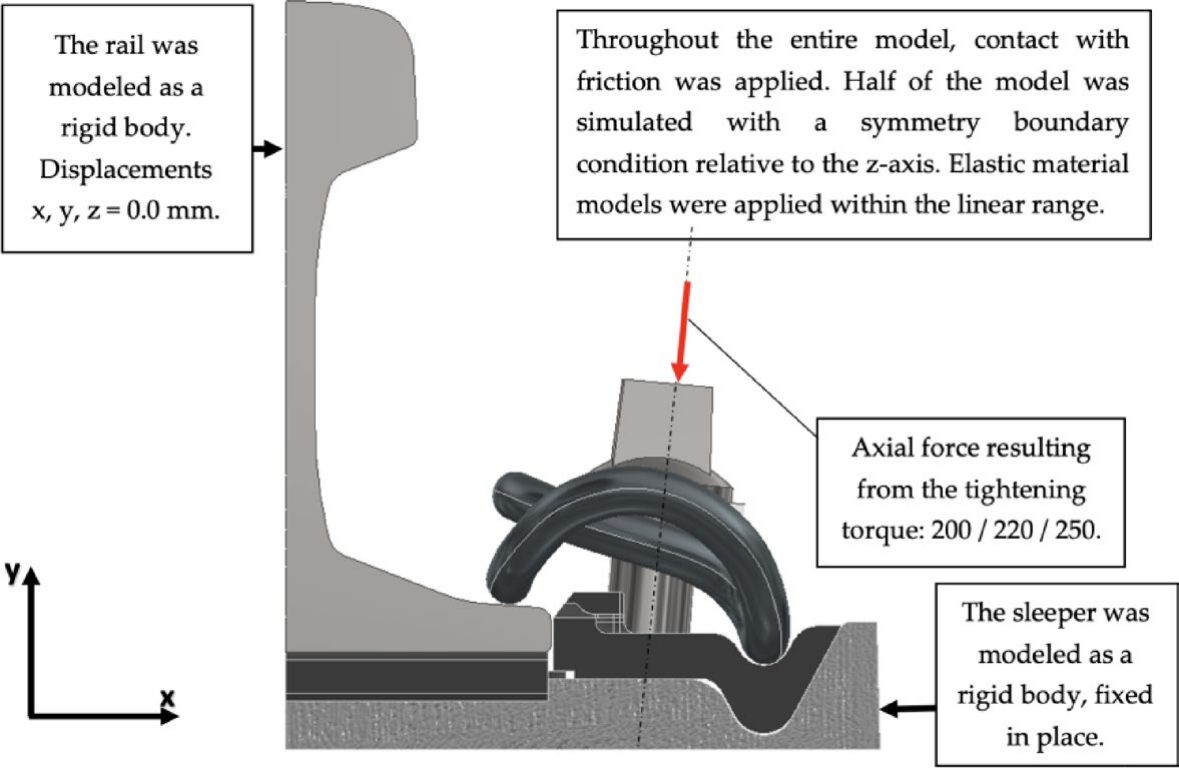
Boundary conditions and loads applied in the FEM analysis.

**Fig. 10:**
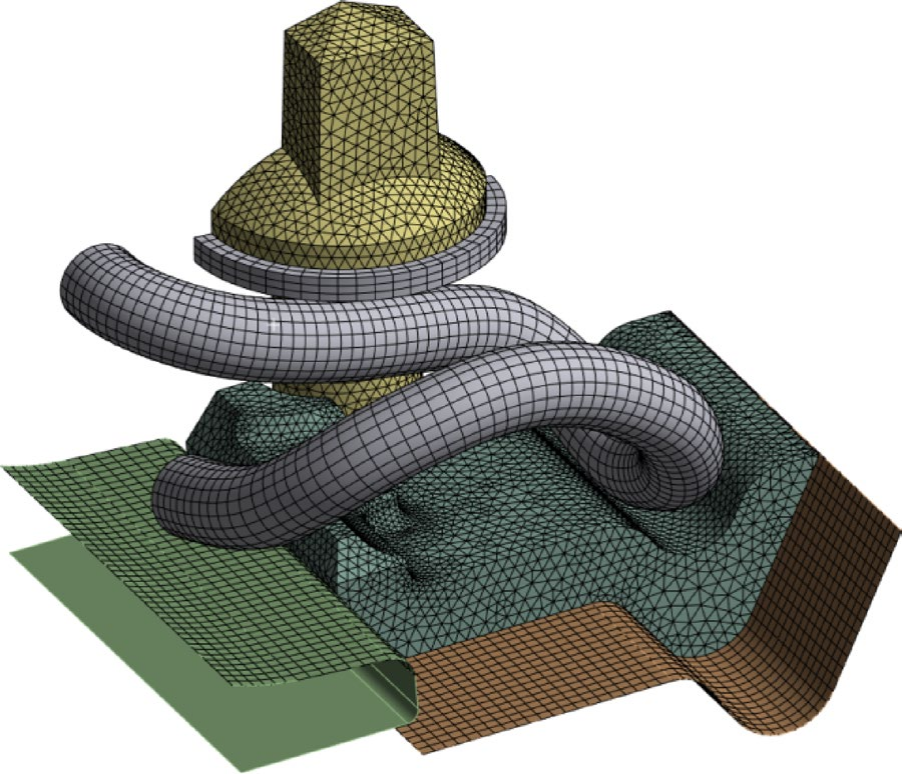
Applied mesh in the FEM analysis. Modelled and analysed in ANSYS 2024 R2 (https://www.ansys.com).

To determine the cause of the influence of the height difference x and to confirm the laboratory tests, Finite Element Method (FEM) simulations of the analysed configurations of the W14 fastening systems were conducted. Figure 9 shows the computational model along with the boundary conditions. The applied mesh is presented in the Fig. 10. The rail and sleeper were modeled as a rigid body.

It was assumed that 200Nm corresponds to the approximated 18kN of clamping force for the most stiff pad – Zw 687a. For higher values the linear relationship between force and torque was applied. The force was applied by bolt pretension function on the core of screw. Applied material models are linear. However the nonlinear contact (finite sliding) and nonlinear geometry were applied (large deflection). Table 3. presents applied material properties. The friction coefficients used in analysis are presented in Table 4.

**Table 3:** Applied material properties.

Name	Young’s Modulus	Poisson’s ratio
	[GPa]	[-]
Clip	210	0.3
Screw	210	0.3
Wfp	5.6	0.35
Sleeper	rigid body approach	
Rail		

**Table 4:** Applied friction coefficients between components.

Component 1	Component 2	Friction coefficient
Clip	Screw	0.0
Clip	Rail	0.4
Wfp	Clip	0.4
Wfp	Concrete	0.4
Washer	Screw	0.6
Clip	Washer	0.6

**Fig. 11:**
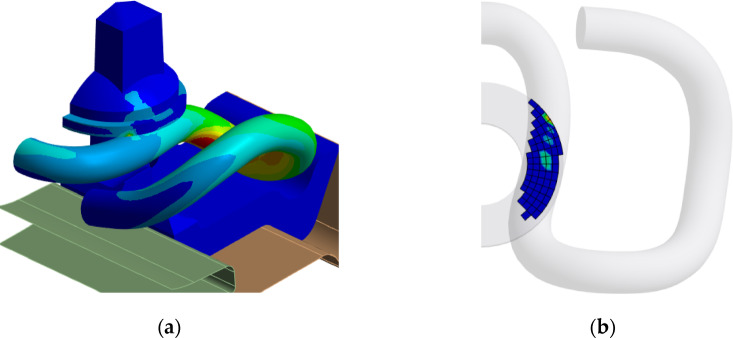
Sample stress distribution (**a**) and pressure at the contact (**b**) for the variant with dimension x = 6 mm and screw tightening torque of 200 Nm and rail clamp SKL 14. Modelled and analysed in ANSYS 2024 R2 (https://www.ansys.com).

Figure 11a presents an exemplary distribution of Von Mises stresses representing the overall stress state for the configuration with a dimension x = 6 mm and a tightening torque of 200 Nm. Figure 11b shows the pressure distribution at the contact between the washer under the screw head and the rail clamp. This distribution is the intermediate cause of changing clamping force values depending.

of rail pads height.

**Fig. 12:**
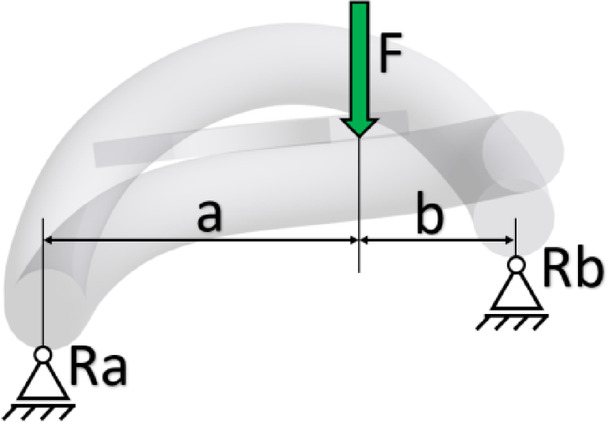
Diagram illustrating the generation of torsional moment on the rail clamp.

The contact reaction forces between the washer under the screw head and the rail clamp were replaced by the resultant force F. Reducing the analysed dimension x increases the lever arm a, generating.

a higher torsional moment around the support Ra (Fig. 12). The change in lever arm a directly affects the value of the reaction force at support Rb. To determine the torsional moment, a simulation was performed considering the change in height and type of rail pads to determine the lever arm a, for each of the considered cases.

**Fig. 13:**
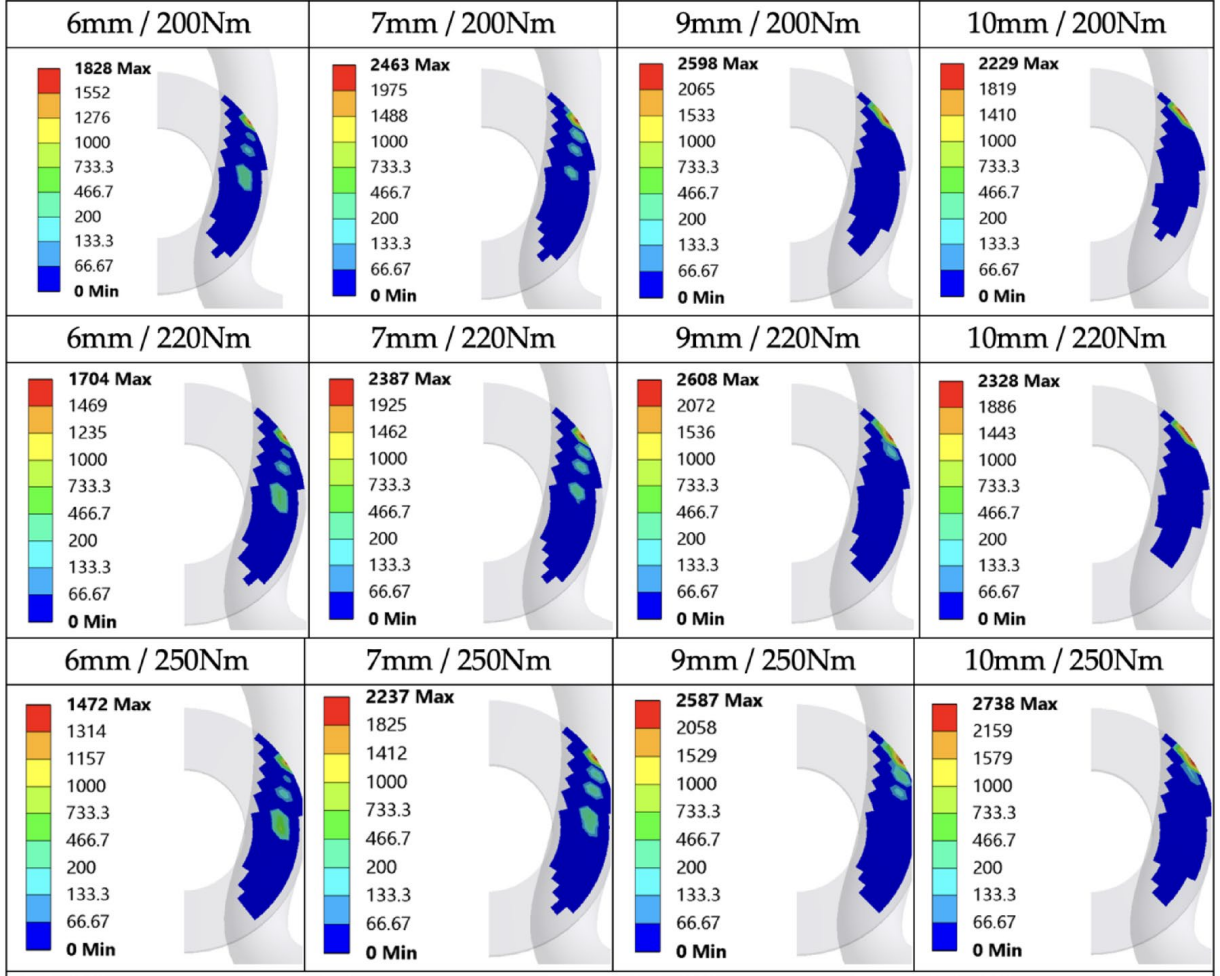
Maps of reaction forces at the contact between the washer under the screw head and the rail clamp SKL 14.

**Fig. 14:**
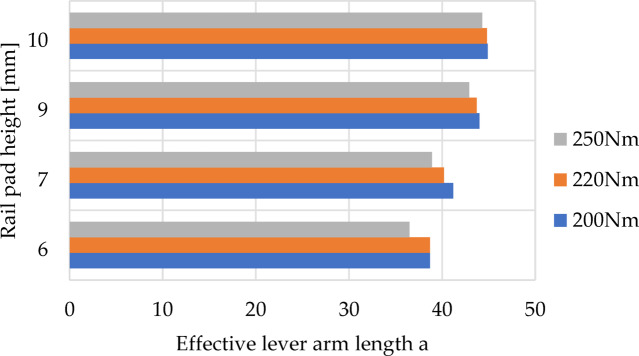
Relationship between the length of the effective lever arm a, and the rail pad height and tightening torque for rail clamp SKL 14.

**Fig. 15:**
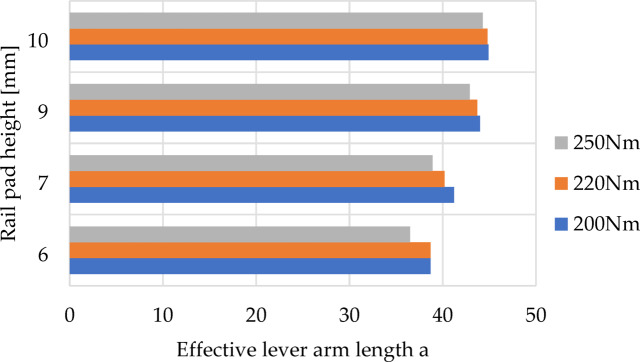
Relationship between the length of the effective lever arm a, and the rail pad height and tightening torque for rail clamp SKL 14RT.

The results of the calculations are maps of reaction forces at the contact between the washer under the screw head and the rail clamp (Fig. 13). Additionally, Figs. 14 and 15 depict the relationship between the length of lever arm a, and the height of the rail pad and the tightening torque, respectively, for rail clamp SKL 14 and rail clamp SKL 14RT.

The results obtained through finite element method simulations confirm the relationship between the variation of dimension x and the clamping force. Increasing the thickness of the rail pad, and consequently increasing the lever arm a, consistently leads to an increase of the clamping force of the fastening system. From the analysis of all obtained data, it is evident that in each analysed variant, the clamping force increases with an increase in the tightening torque and the thickness of the rail pads (dimension x). Higher clamping force values were also obtained for SKL 14 RT rail clamps (with increased rod diameter). Regarding longitudinal resistance, the highest values were obtained for rail pads with thicknesses of 9 and 10 mm and lower static stiffness. Increasing the tightening torque to the level of 500 Nm does not affect the increase in the clamping force of the fastening system.

## Conclusions

The research conducted on the selected parameters of the W14 fastening system indicates that the proper installation of the fastening system significantly influences the obtained results. According to the manufacturer recommendations, screw tightening during operation should be performed with.

a torque of 250 Nm. However, achieving the correct clamping force requires adjusting the torque, considering the impact of rail pad height. The diameter of the rod used for the rail clamp has.

significant effects on the analysed parameters of the fastening systems. It should be noted that a key parameter influencing the increase in clamping force in the fastening system is the height and static stiffness of the rail pad. Changing the height of the rail pad modifies the length of the lever arm on which the force acts which in turn directly influences the actual clamping force that can be achieved. Further investigation of this issue is warranted because uneven stress on the rail pad may lead to significant plastic deformations. Continued research is advisable to examine the effects of time and operating conditions on the clamping force. According to guidelines and requirements, the aging effect of changes in clamping force and other fastening system parameters can significantly affect the longevity of the fastening system and its impact on the surrounding environment. This variations may have a considerable impact on damping effectiveness and, consequently, on environmental impact in terms of track durability and noise comfort or lack thereof, directly affecting people. In particular, the influence of lateral forces acting on the rail head should be considered, as indicated, for example,

in^17^, where extreme cases may result in rail inclination, ultimately leading to increased forces in the contact between the washer under the screw head and the spring. Increasing the plasticity zone may alter the force distribution in the contact, resulting in a change in clamping force. Therefore, it is necessary to verify whether increasing the thickness of the rail pad is a reliable and long-lasting method for partially increasing the clamping force.

## Data Availability

The datasets used and/or analysed during the current study available from the corresponding author on reasonable request.
